# Genome-wide identification and expression analysis of WRKY family genes under soft rot in Chinese cabbage

**DOI:** 10.3389/fgene.2022.958769

**Published:** 2022-09-26

**Authors:** Jinghui Yan, Xinle Yu, Wei Ma, Xiaoxue Sun, Yunjia Ge, Xiaonan Yue, Jing Han, Jianjun Zhao, Yin Lu, Mengyang Liu

**Affiliations:** ^1^ Collaborative Innovation Center of Vegetable Industry in Hebei, College of Horticulture, Hebei Agricultural University, Baoding, China; ^2^ Key Laboratory of Vegetable Germplasm Innovation and Utilization of Hebei, Baoding, China

**Keywords:** Chinese cabbage (*B. rapa* ssp. *pekinensis*), soft rot, WRKY transcription factor, transcriptome, expression profile difference

## Abstract

Complex transcriptional networks regulate plant defense against pathogen attack, and plant transcription factors act as key regulators of the plant immune responses. The differences between transcription factor expression and regulation in Chinese cabbage soft rot (*Pectobacterium carotovorum*; Pc) have not been revealed. In this study, a total of 148 putative Chinese cabbage WRKY genes (BrWRKYs) were identified from the Chinese cabbage genome (v3.0). These genes were divided into seven subgroups (groups I, IIa–e, and III) based on phylogenomic analysis, with distinct motif compositions in each subgroup. Time-series RNA-seq was carried out to elucidate the dynamic expression patterns of the BrWRKYs on the resistant mutant (sr) and the susceptible wild-type (inbred WT) challenged by Pc. Transcriptional analysis showed that 48 WRKY transcription genes at 0–24 hpi were significantly upregulated in sr under soft rot stress. At the 12-h post-inoculation critical time point, we identified three specifically upregulated genes and two downregulated genes in the resistant mutant, which may provide potential applications for genetic improvement against soft rot. The findings improved our understanding of the WRKY-mediated soft rot stress response regulation in Chinese cabbage. The study thus lays a foundation for the genetic improvement of soft rot resistance.

## 1 Introduction

Plants have an innate immune system to avoid the invasion of pathogens (Jones and Dangl, 2006; Garner et al., 2016). Various transcription factors (TFs) *via* transcription networks regulate this plant immune system at the transcriptional level (Kunkel and Brooks, 2002; Dodds and Rathjen, 2010; Birkenbihl et al., 2017). Numerous studies have shown that few plant TF families, such as AP2/ERF, bHLH, ZIM, NAC, JAZ, and WRKY, are the key regulators of defense responses (Tsuda and Somssich, 2015). The WRKY gene family is one of the most widely studied TF families of higher plants. In recent years, it has been reported that WRKY transcription factors are involved in disease resistance such as in tomatoes (Chinnapandi et al., 2019), pepper (Dang et al., 2013; Dang et al., 2014; Cheng et al., 2020), potato (Yogendra et al., 2013)and other crops (Liu et al., 2007; Ko et al., 2015). Studies have shown that WRKY protein is involved in a variety of plant disease resistance responses from basic immunity to acquired resistance, and it participates in plant defense responses to pathogens by regulating a multi-pathway and multi-level disease resistance signal pathways (Eulgem and Somssich., 2007; Phukan et al., 2016; Birkenbihl et al., 2018). Although research has been extensive and in-depth, the *Brassica* WRKY family has been comprehensively identified and analyzed (He et al., 2016; Kayum et al., 2015; Tang et al., 2014). However, on the one hand, these studies are relatively early and cannot match the update of the genome version. On the other hand, its transcriptional expression is more related to the expression under abiotic stress, and there is a relative lack of research on the expression and regulation of WRKY transcription factors under biological stress of *Brassica* plants, especially under the stress of Chinese cabbage soft rot.

Chinese cabbage (*Brassica rapa* ssp. *pekinensis*) is an important vegetable, which is very popular in Asia. Soft rot caused by the pathogen *Pectobacterium carotovorum* (Pc) is one of the three major diseases of Chinese cabbage. Pc is a necrotrophic bacterium with a wide host range and usually stays on the plant surface and soil (Reverchon et al., 2016; Rabia et al., 2020). It infects the host through natural pores on the plant surface or wounds. It secretes hydrolases that degrade plant cell walls, extracts nutrients from plant tissues to support their own growth and reproduction, and when environmental conditions such as moisture, oxygen, and temperature are conducive, it further infects and enters plants, causing diseases. However, this is very complicated with regard to resistance to Pc in Chinese cabbage. Some studies have preliminarily clarified the hypothetical molecular mechanism of Chinese cabbage’s resistance to Pc (Liu et al., 2019), but the molecular basis of our resistance to this soft rot plant pathogen, especially the role of transcription factors, remains to be explored. Some studies have shown that WRKY transcription factors play an important role in Pc stress. On the other hand, WRKY70 plays a key role in balancing SA-dependent and JA-dependent signal defense Pc (Li et al., 2004). On the other hand, WRKY75 positively regulates JA- or SA-dependent defenses, and WRKY33 is a positive regulator of JA-dependent genes, which plays an important role in resistance to Pc infection (Zheng et al., 2006; Birkenbihl et al., 2012; Choi et al., 2014). In summary, the analysis of the WRKY transcription level of Chinese cabbage inoculated with Pc is an important way to understand the response mechanism of soft rot and to select resistant varieties.

In this study, we performed another characterization and analysis of the WRKY gene family using the Chinese cabbage v3.0 genome. We also analyzed the genome-wide identification of the WRKY gene family such as the chromosomal location, gene structures and protein conserved sequence alignment, and conserved domains. We also integrated transcriptional regulation and expression analyses of the WRKY gene: time-series RNA-seq was carried out to elucidate the dynamic expression patterns of the BrWRKYs under Pc in Chinese cabbage. Also, we screened the key BrWRKYs of Pc resistance by differential analysis and expression analysis. This discovery will enrich our understanding of the role of WRKY in soft rot disease resistance and provide novel insights on approaches to improve disease resistance in Chinese cabbages.

## 2 Results

### 2.1 Identification of WRKY in Chinese cabbage and basic structure analysis

**FIGURE 1:**
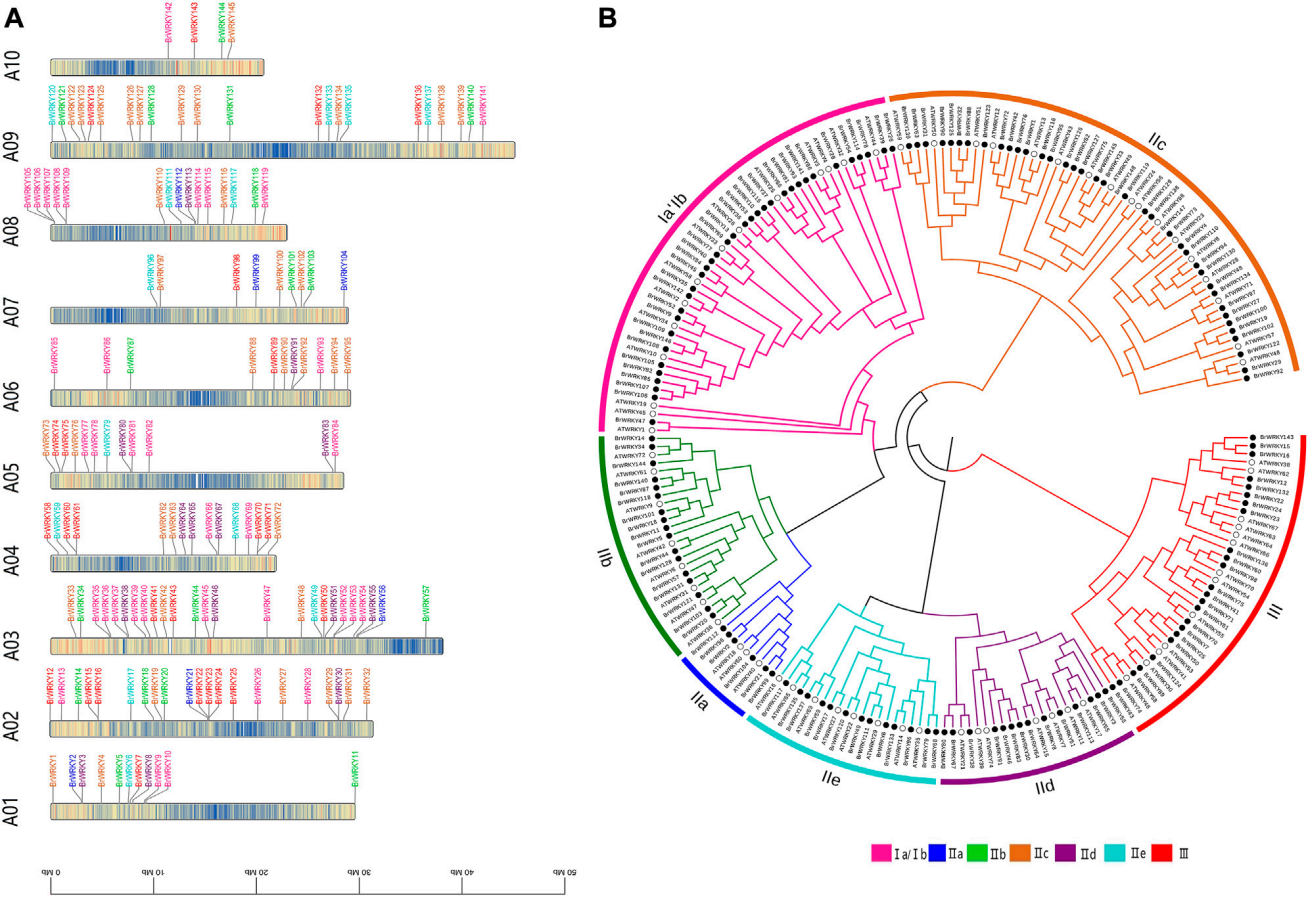
Chromosomal location and phylogenetic tree analysis of BrWRKY family members. **(A)** Classification and distribution of the 145 BrWRKY genes in Chinese cabbage identified in this study on 10 chromosomes and visualized by TBtools (another three located in subgenomes are not shown). **(B)** Multiple alignment of the 148 BrWRKYs and 71 AtWRKYs from *Arabidopsis thaliana*, and the phylogenetic tree was constructed by MEGA based on the neighbor-joining (NJ) method.

We identified all members of the Chinese cabbage WRKY TFs. According to the Chinese cabbage Brassica_rapa. Brapa 3.0 genome version PEP (V 3.0) (BRAD V3.0, http://brassicadb.org/brad/) (Chen et al., 2022), 148 Chinese cabbage WRKY genes were identified. Due to the update of the genome version, there is a quantitative difference between this (148) and the results (145) found by predecessors (Tang et al., 2014). Concretely, BrWRKY53 (Bra019123) and BrWRKY58 (Bra023983) are the same loci that were merged into BrWRKY56 (BraA03g056960.3C); BrWRKY41 (Bra000202) and BrWRKY71 (Bra016975) are the same loci that were merged into BrWRKY70 (BraA04g028830.3C); and we re-identified BrWRKY26 (BraA02g030090.3C), BrWRKY108 (BraA08g002070.3C), BrWRKY109 (BraA08g002100.3C), and BrWRKY146 (BraAnng003190.3C) as four BrWRKYs. To avoid confusion, we designated these WRKY genes from BrWRKY1 to BrWRKY148 based on their chromosomal location. The main WRKY genetic characteristics of *B. rapa* are summarized in additional file 1, including the gene names, gene ID, chromosome location, group, full length of cDNA, molecular weights, isoelectric points, instability index, aliphatic index, and grand average of hydropathicity. For all BrWRKYs, the molecular weights range from 13,583.14 Da to 98,194.09 Da, the full length of cDNA from 117 bp to 868 bp, and the isoelectric points from 4.85 to 9.92, as shown in additional file 1. The location of the WRKY genes on the chromosomes is shown in Figure 1A. Except for the three members on the scaffold, the other 145 BrWRKYs were mapped to A01–A10 chromosomes. Most WRKY genes (98/148) were on chromosomes 2, 3, 4, 8, and 9. The analysis revealed the largest number of TFs on chromosome A03 (25/148 members; 17%) and the least number of TFs on chromosome A10 (4/148, <3%).

To better understand the phylogenetic relationships of WRKY genes in Chinese cabbage, *Arabidopsis*, a neighbor-joining (NJ) phylogenetic tree was built based on the multiple sequence alignment of the 148 BrWRKYs and 71 AtWRKYs from *Arabidopsis thaliana* (Figure 1B). The TAIR (http://www.arabidopsis.org/) website has announced three major categories and five subcategories of the *Arabidopsis* WRKY family. According to the number of WRKY domains and the composition of the zinc finger structure, the 148 BrWRKYs are divided into three major categories and seven large subcategories: I, IIa, IIb, IIc, IId, IIe, and III which had 36, 6, 17, 38, 14, 13, and 24 members, respectively. Typically, the DNA-binding activity of WRKY transcription factors depends on the conserved sequence of WRKYGQK. Therefore, we performed gene structure conserved domain and motif analysis of BrWRKYs and constructed an ML phylogenetic tree to reveal the relationship between the WRKY family structure and evolution. Additional file 2 shows that all BrWRKYs contain at least one motif1 (WRKY) domain, and each subgroup has its canonical motif composition; these motifs are essential for the function of WRKY proteins.

### 2.2 Identification of cis-acting elements of BrWRKY promoters

**FIGURE 2:**
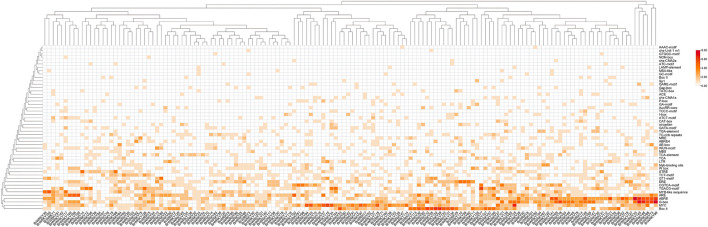
All 148 BrWRKYs cis-element originals were analyzed as a cluster heatmap. The 2000-bp upstream region of the BrWRKYs was extracted as the promoter sequence and submitted to the PlantCARE database (http://bioinformatics.psb.ugent.be/). In this way, the cis-acting element of the Chinese cabbage WRKY family genes was predicted.

We first analyzed the cis-acting elements of the promoter sequences 2000 bp upstream of all these BrWRKYs in Chinese cabbages. Our analysis revealed that the whole family of BrWRKY has multiple cis-acting elements, especially hormone-related elements (the ABA-responsive element ABRE, jasmonic acid-responsive element TGACG, salicylic acid-responsive element CCATCTTTTT, gibberellin-responsive element TCTGTTG, and ethylene-responsive element ERE) and stress-related elements (the stress response element TC-rich repeats, low-temperature response element LTR, drought response element MBS, and wound response element), which respond to biotic and abiotic stresses (Figure 2). These BrWRKYs’ cis-acting elements combine with various stress-related trans-acting factors to regulate gene expression and response of stress resistance genes in Chinese cabbage.

### 2.3 Analysis of BrWRKY expression in Chinese cabbage

Expression differences of BrWRKY genes in Chinese cabbage under Pc and the raw RNA-seq data used here were generated from a previous study conducted in our research group (Liu et al., 2019). Based on the published transcriptome data, we reconducted the quality control and mapped it to the *B. rapa* reference genome (v3.0) in the Brassica database (BRAD V3.0, http://brassicadb.org/brad/) (Chen et al., 2022). We further analyzed the expression of BrWRKY genes to soft rot stress and identified a group of abnormal BrWRKY genes of the two lines.

#### 2.3.1 Global analysis of TF DEGs in response of Chinese cabbage to Pc infection

**FIGURE 3:**
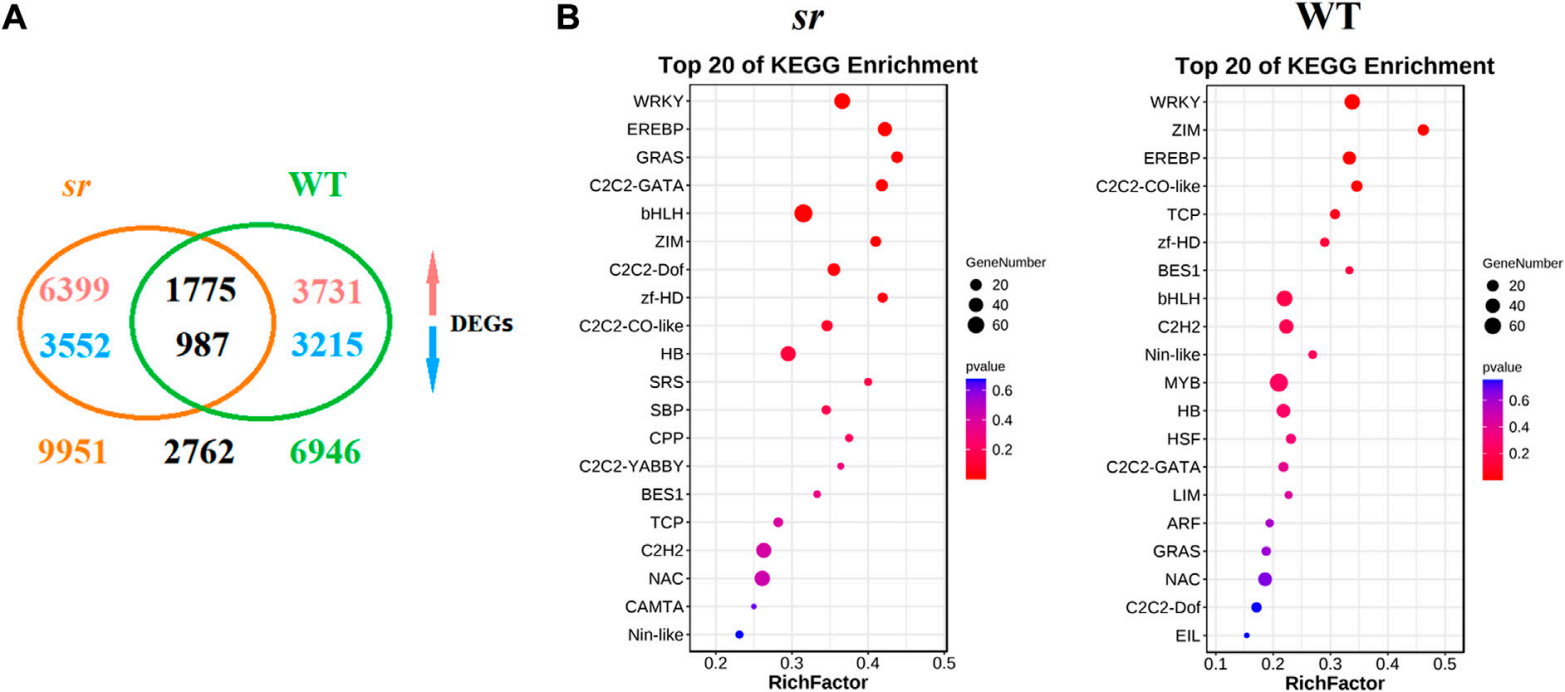
DEG identification and enrichment analysis between susceptible WT and resistant sr after Pc. **(A)** In total, 6,945 and 9,951 differentially expressed genes (DEGs) were identified from WT and sr, respectively, and 2,762 DEGs were commonly identified in both lines. The upward arrow represents the upregulated expression. The downward arrow represents the downregulated expression. **(B)** Enrichment analysis of upregulated TF DEGs between Chinese cabbage lines WT and sr after Pc infection. To gain insights into the 148 TF families in response to Pc infection, enrichment analysis was performed using all TF DEGs in the two lines. Among all these TF families, the WRKY family was most significantly enriched both in WT and sr. The enrichment analysis was performed by the ggplot2 package (http://had.co.nz/ggplot2/).

We identified differentially expressed genes (DEGs, *p*-value < 0.05 and FDR ≤ 0.01) in transcriptome data, of which we identified 6,945 DEGs in WT, 3,731 were upregulated, and 3,215 were downregulated. Meanwhile, 9,951 DEGs were identified in sr, of which 6,399 were upregulated and 3,552 were downregulated (Figure 3A). Among these identified DEGs, we identified 3,295 TFs and classified them into 45 families based on the Plant Transcription Factor database (PlantTFDB V5.0) (Ko et al., 2015). We identified 839 differentially expressed TF genes in the resistant mutant sr after Pc inoculation, accounting for approximately 25.5% of all the identified TF genes. In comparison, 664 TF genes showed significant changes in the susceptible WT. These results indicate substantial transcriptional changes during Pc infection in both sr and WT, with more DEGs and differentially expressed TFs in sr. Furthermore, to understand the role of these 45 TF families in response to Pc infection, we performed enrichment analysis using the identified TF DEGs in the two lines. The analysis revealed that the top five enrichment values of TFs according to the *p*-value were WRKY, AP2-ERFBP, ZIM, ZF-HD, and bHLH in all the identified TF genes. The WRKY TF had an extremely significant enrichment *p*-value in both sr and WT and significantly enriched DEGs in both lines among the different TF families (Figure 3B). Thus, our comparative transcriptome analysis indicates the putative role of WRKY in regulating the host’s immune response to Pc. In addition, we also made an in-depth analysis of Chinese cabbage WRKY TFs in order to study the molecular mechanism and resistance genes of Chinese cabbage resistance to Pc.

#### 2.3.2 Response of BrWRKYs to soft rot stress

**FIGURE 4:**
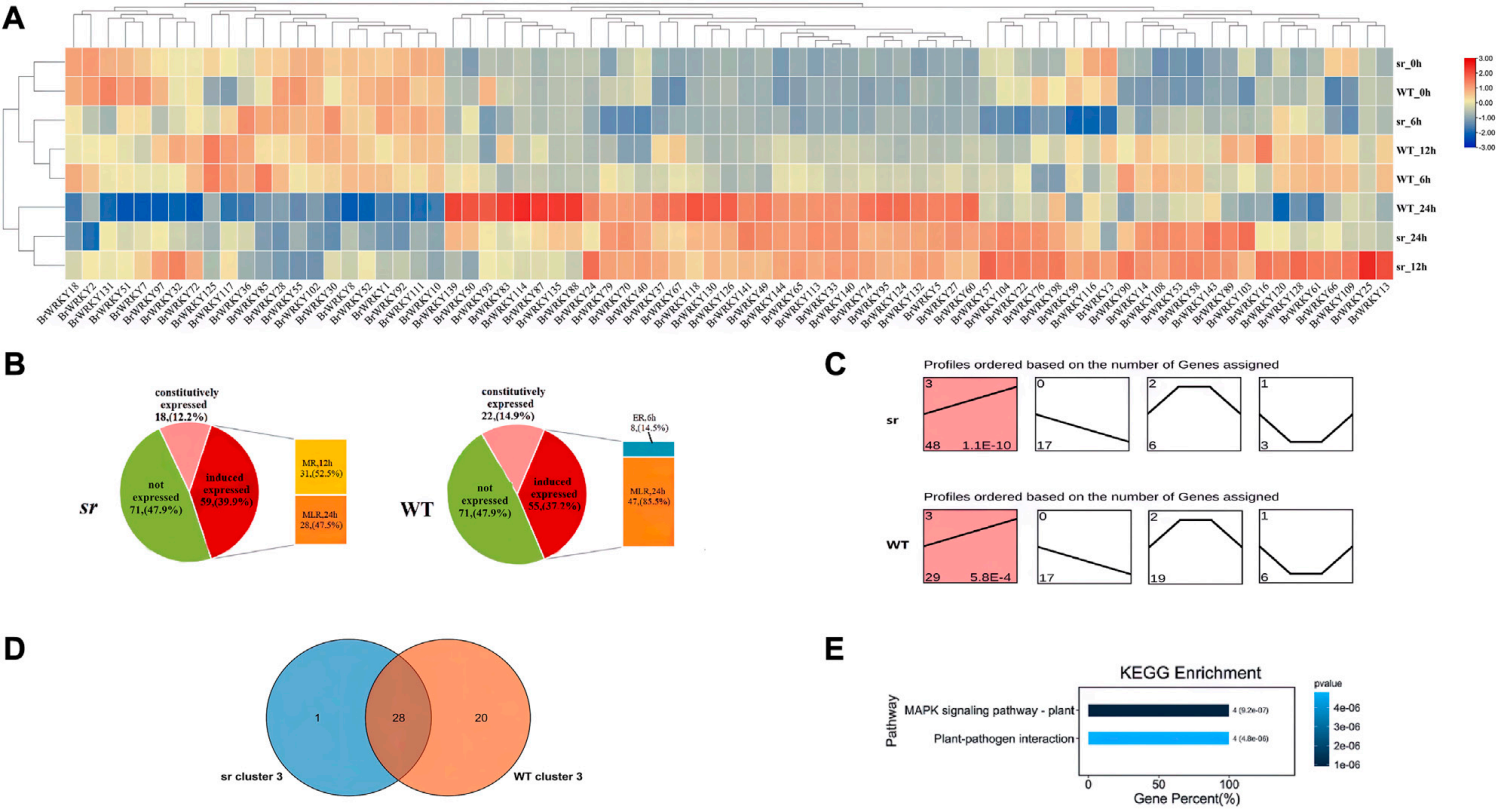
Expression response of BrWRKYs to Pc inoculation. **(A)** Heatmap of WRKY family members in Chinese cabbage response to Pc infection. **(B)** Analysis of the expression trend of BrWRKYs under soft rot stress. These BrWRKYs can be divided into early response (6 hpi, ER), middle response (12 hpi, MR), and middle and late response (24 hpi, MLR) genes. **(C)** Transcriptional patterns of the expression of 77 BrWRKY members in WT and sr are shown in **(C)**. **(D)** Relative to clusters 0–2, cluster 3 is the dominant type, with the largest number, and the *p*-value is more significant, including sr 48 and WT 29 BrWRKYs, and 28 were identified in both lines and are shown in the Venn diagram. **(E)** KEGG functional enrichment analysis for cluster 3. Cluster 3 expression was upregulated at 0–24 hpi of soft rot stress.

We analyzed the time-course transcriptome data to determine the response of WRKY genes to soft rot stress. The analysis detected no expression for 71 of 148 BrWRKYs, while the expression of 77 BrWRKYs in response to Pc infection is shown in the heatmap; eight samples (WT and sr at 0,6,12, and 24 hpi) were grouped into two subgroups by a heatmap (Figure 4A). The first subgroups mainly consist of five samples, including WT and sr at 0 and 6 hpi, and WT at 12 hpi. The other subgroups have three samples, including WT at 24 hpi, and sr at 12 and 24 hpi. We can find that sr 12 hpi has more similar expressions to WT and sr at 24 hpi, which means that the defense response of sr to Pc may start from 12 hpi and continue to 24 hpi. Furthermore, according to the difference analysis of detected gene expression, 59 BrWRKYs were differentially expressed in the sr, while only 55 genes were differentially expressed in WT. The responses of these differentially expressed genes to Pc inoculation were significantly different at different time points. According to the time when BrWRKYs begin to respond to Pc, it can be divided into early response (6 hpi, ER), middle response (12 hpi, MR), and middle and late response (24 hpi, MLR) genes (Figure 4B). Among the 59 BrWRKYs expressed in sr, 0 were ER genes, 31 were MR genes, and 28 were MLR genes. In the WT, 8 were ER genes, 0 were MR genes, and 47 were MLR genes. Based on the aforementioned results, there were significant differences in the expression patterns of BrWRKY transcription factors between the two lines. We speculate that 12 hpi is the key time point for transcriptional expression of the WRKY family members of Chinese cabbage under Pc stress, which may be the reason for the difference in resistance to soft rot in Chinese cabbage.

We clustered the expression of BrWRKYs to study its trend analysis and constructed four clusters (cluster_0–3) based on a K-means clustering method (Figures 4C,D). Cluster_0 containing 17 members in WT, the same as in sr, decreased significantly in response to Pc infection at 0–24 hpi. Cluster_1 containing 6 members in WT, but three members in sr, had 0–6 hpi downregulated genes, 6–12 hpi stably expressed genes, and 12–24 hpi upregulated genes. Cluster_2 containing 19 members in WT, but 6 members in sr, had the exact opposite expression pattern to cluster_1. However, these clusters do not seem to be the response pattern of Chinese cabbage defense against Pc. Relative to cluster_0–2, cluster_3 is the dominant type, with the largest number, and the *p*-value is more significant. It contains 29 (*p* = 5.8 × 10–4) members in WT, but 48 members in sr (*p* = 1.1 × 10–10), and the expression was upregulated at 0–24 hpi of soft rot stress. We also found that the sr had relatively more upregulated genes in cluster_3, which may be another reason for the difference of resistance to soft rot between the two lines. On the other hand, the KEGG functional enrichment analysis for cluster_3, which was mapped to two KEGG pathways and those KEGG pathways that were most significantly identified, included the MAPK signaling pathway–plant and plant–pathogen interaction (Figure 4E).

#### 2.3.3 Analysis of the differential expression of BrWRKYs under soft rot stress between the two lines

**FIGURE 5:**
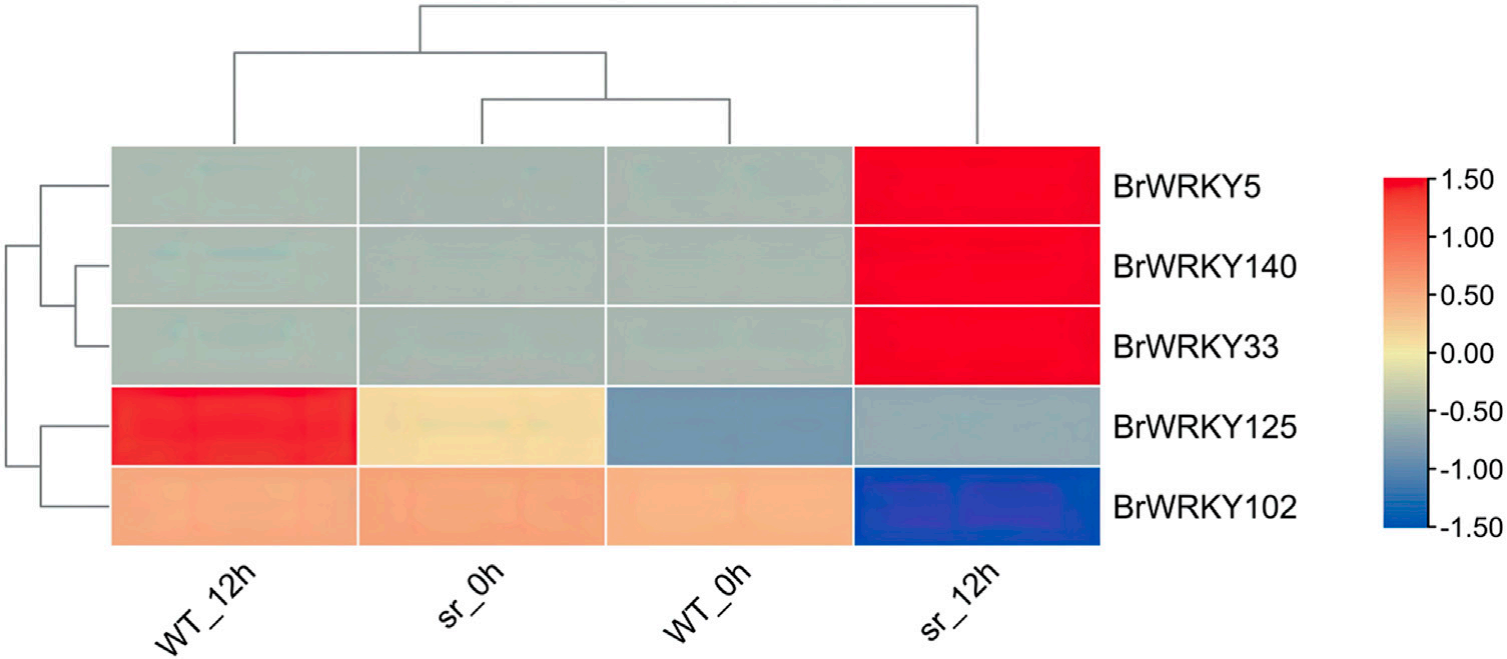
Heatmap of differential expression of BrWRKYs between susceptible WT and resistant sr at 12 hpi. Tests for pairwise differential expression were performed in the DESeq2 R package. The resulting *p*-values were adjusted to control the false discovery rate (FDR), with genes having *p*-values < 0.05, |FoldChange| > 2, and FDR ≤ 0.01 considered to be differentially expressed genes (DEGs).

In depth, we analyzed the differential expression of the two lines at different time points; only five BrWRKYs (three upregulated and two downregulated) were identified at 12 hpi (Figure 5), for which the BrWRKY5 were 140, 33, 125, and 102. Therefore, these BrWRKYs differentially expressed at the critical time point of 12 hpi may be one of the reasons for the resistance of the two lines to soft rot.

## 3 Discussion

WRKYs are one of the most prominent families of TFs in higher plants that play key roles in response to biotic and abiotic stresses (Cheong et al., 2002; Rushton et al., 2010; Wang et al., 2011). A number of WRKY TFs have been identified with genome-wide analysis, for example, *Arabidopsis*, rice, and other plants and even Chinese cabbage (*Brassica rapa* ssp. *pekinensis*), such as the Chinese cabbage Brassica_rapa. Brapa_1.0 genome. Despite the continuous improvement of the reference genomes of Brassica_rapa. Brapa, genome-wide analysis of WRKY TFs cannot match the update of the genome version. According to the Chinese cabbage Brassica_rapa. Brapa_3.0 genome version PEP (V 3.0) (Chen et al., 2022), the study identified 148 WRKY TFs in Chinese cabbage. Also, the number of WRKYs in Brapa_3.0 was more than that of Brapa_1.0, while in Chinese cabbage Brapa_1.0 145 WRKYs were found (Tang et al., 2014). Due to the rapid advances of sequencing technology in recent years, high-quality reference genome sequences of BRAD have been either decoded or upgraded, which guarantee better accuracy of gene annotation. Among the Chinese cabbage WRKY families identified by us, there are four BrWRKYs (BrWRKY26-BraA02 g030090.3C, BrWRKY108-BraA08 g002070.3C, BrWRKY109-BraA08 g002100.3C, and BrWRKY146-BraAnng003190.3C) that have not been identified in Brapa_1.0. Also, two pairs of previously identified BrWRKYs (BrWRKY53-Bra019123 and BrWRKY58-Bra023983, and BrWRKY41-Bra000202 and BrWRKY71-Bra016975) are coincident, and they are combined into two BrWRKYs (BrWRKY56-BraA03 g056960.3C and BrWRKY70-BraA04g028830.3C). In addition, the number of WRKY TFs in Chinese cabbage (148) is much more than that in *Arabidopsis thaliana* (74). Both *Arabidopsis* and Chinese cabbage belong to the Brassicaceae family, and the Chinese cabbage is also a subspecies of *B. rapa*, which has undergone polyploidization, leading to additional whole-genome triplication (Cai et al., 2021). The BrWRKY gene family was expanded by 2-fold, and two or three copies were homologous to one AtWRKY protein. However, nine AtWRKYs were missing in the Chinese cabbage: AtWRKY5, 19, 37, 43, 52, 60, 63, 64, and 73 (Figure 1B). Also, most BrWRKY genes (98/148) were concentrated on chromosomes 2, 3, 4, 8, and 9. All these BrWRKYs were segmentally duplicated and unevenly distributed on the genome, which played a role in genomic rearrangement and diversification.

WRKY family TFs were divided into seven subfamilies according to their conservative domains, including I, IIa–e, and III (Eulgem et al., 2000). The WRKY family in Chinese cabbage Brassica_rapa. Brapa_3.0 also consisted of these seven parts: group I with a 2.2-fold expansion compared to *Arabidopsis* and containing 36 members but 32 members in Brapa_1.0, group IIa–e with a 2.0-fold expansion and 88 members but 89 members in Brapa_1.0, and group III with a 1.7-fold expansion and 24 members but 25 members in Brapa_1.0 (Additional file 1). Also, group II has the largest number, while in *Arabidopsis*, group I has the largest number, and in rice, group III has the largest number. However, most BrWRKYs proteins of the same subfamily shared not only the relatively similar conservative domain but also similar gene structures (Additional file 2). On the other hand, abundant cis-acting elements have been found in the upstream of WRKY transcription factors in Chinese cabbage, which are related to abscisic acid, jasmonic acid, salicylic acid, ethylene, and a variety of stresses. These BrWRKY’s cis-acting elements combine with various stress-related trans-acting factors to regulate gene expression and response of stress resistance genes in Chinese cabbage.

We detected the expression of 77 BrWRKYs in Chinese cabbage leaves under soft rot stress, and the expression of the remaining 71 BrWRKY genes seemed to be tissue-specific, consistent with the expression profiling of BrWRKYs in different tissues (Kayum et al., 2015). Susceptible WT and resistant sr were used as materials, and we found 59 and 55 DEGs, accounting for 35.8% and 28.4% of all WRKY factors. These findings indicate an apparent response to stress involving WRKY TFs, consistent with the functional study of WRKY family members in the infection response of *Phytophthora capsici* (Cheng et al., 2020). We compared the expression of WRKYs in the two lines at 0, 6, 12, and 24 hpi with soft rot and found that sr has more responsive genes in 12 hpi. Also, reflected in the results of heatmap clustering, the expression pattern of sr 12 hpi is more similar to sr and WT at 24 hpi. It is inferred that 12 hpi may be a crucial time point for the transcriptional expression of WRKY family members of Chinese cabbage under Pc stress, consistent with the previous studies on Chinese cabbage (Liu et al., 2019). In the expression trend analysis, the two lines had more upregulated expression (cluster 3), and the expression was upregulated at 0–24 hpi of soft rot stress. We used the different degrees of sensitivity of the two materials to soft rot for comparison; the sr had relatively more upregulated genes in cluster 3. Also, the same thing is that sr has a higher expression level in 12 hpi than in WT when we analyzed the differential expression of the two lines at different time points, which may be another reason for the difference of resistance to soft rot between the two lines. Previous studies have found that some WRKY genes play an important role in the response of Chinese cabbage to Pc stress (Liu et al., 2019). In its research, the WRKY33 transcription factor is the downstream gene of plant resistance to necrotizing pathogens, which was identified at 12 hpi in the response of Chinese cabbage to Pc resistance. Also, WRKY70 is the core component of SA signaling, WRKY70 is upregulated in the response of Chinese cabbage to Pc resistance to promote the expression of downstream genes. However, we analyzed the differential expression of BrWRKYs in the two lines under soft rot stress and obtained different results. Five BrWRKYs (three upregulated and two downregulated) were identified at 12 hpi, among which BrWRKY5-BraA01g012730.3C, BrWRKY140-BraA09 g056990.3C, and BrWRKY33-BraA03g005540.3C had obvious response expression in the stress response of the Chinese cabbage soft rot, which was upregulated at 0–24 hpi. Susceptible WT and resistant sr were used as materials, and in 12 hpi-resistant plants, the expression was 3.3-fold, 4.3-fold, and 4.5-fold, respectively, with significant response differences. We infer that AtWRKY31 (BrWRKY5) and AtWRKY75 (BrWRKY33, 140) may be the key genes of Chinese cabbage in response to Pc stress and play an important role.

## 4 Materials and methods

### 4.1 Identification and sequence analysis of WRKY genes in Chinese cabbage

The hidden Markov model (HMM) profile of the WRKY domain (PF03106) was downloaded from the Pfam database (http://pfam.xfam.org/), and preliminary screening of the Chinese cabbage genome (BRAD V3.0, http://brassicadb.org/brad/) was conducted using HMMER (Chen et al., 2022). Then, the candidate sequences were searched twice by BLASTP based on the amino acid sequence of the conserved domain, summarized, and deduplicated. The identity of all the WRKY genes was confirmed by comparing against the Pfam database, conserved domain database (CDD; https://www.ncbi.nlm.nih.gov/cdd/), and SMART database (http://smart.embl.de/smart/batch.pl) to validate the presence of the WRKY domain.

### 4.2 Sequence alignment, phylogenetic, and conserved motif analysis

To study the phylogenetic relationships of BrWRKY proteins and orthologs in *Arabidopsis*, sequences of AtWRKY TFs were retrieved from TAIR (http://www.arabidopsis.org). The phylogenetic tree was constructed by MEGA (v7.0.26) (Kumar et al., 2016) based on the neighbor-joining (NJ) method, with a bootstrap value of 1,000. The BrWRKY protein sequences for conserved motif analysis were analyzed by MEME (https://meme-suite.org/meme/tools/meme, accessed on 10 January 2022) (Bailey et al., 2009) to detect the possible conserved motifs using default parameters; the maximum number of motifs to be identified was defined as 3, and the maximum width was set as 200. The NCBI CD-Search tool was used for recognition and prediction of structural domains. Furthermore, TBtools (V 1.098696) (Chen et al., 2020) was used to visualize the phylogenetic tree, conserved motif map, structural domain distribution map, and gene structure map according to the phylogenetic tree file and MEME file obtained previously and the GFF file and CDS file of the Chinese cabbage genome database.

### 4.3 Chromosomal location and cis-acting elements of WRKY family genes

The chromosomal distribution of BrWRKY genes was mapped according to the physical location and length of chromosomes based on the V3.0 version of the Chinese cabbage genome annotation file (GFF3) and the corresponding genomic DNA sequences.

The 2000-bp upstream region of the BrWRKYs was extracted as the promoter sequence and submitted to the PlantCARE database (Lescot et al., 2002) (http://bioinformatics.psb.ugent.be/). In this way, the cis-acting element of the Chinese cabbage WRKY family genes was predicted and visualized by TBtools (V 1.098696) (Chen et al., 2020).

### 4.4 RNA-seq analysis

Transcriptomic data on Chinese cabbage were obtained from the National Center for Biotechnology Information (NCBI) publicly accessible database (Accession number: GSE209906). Also, the raw RNA-seq data used here were generated for a previous study conducted in our research group (Liu et al., 2019). The RNA from three biological replicates of Chinese cabbage soft rot-resistant mutant sr and WT at 0, 6, 12, and 24 hpi (24 samples) were extracted using the TRIzol reagent (Invitrogen, United States), according to the manufacturer’s instructions (Wang et al., 2009; Lu et al., 2014; Lu et al., 2016). We reconducted the quality control and trimming to filter the adapter sequences and unknown/low-quality reads with fastp (Chen et al., 2018). The clean data were mapped to the *B. rapa* reference genome (v3.0) in the Brassica database (BRAD). After filtering the reads, 179.17 Gb of high-quality sequences (> 96% of the raw reads) were obtained from the 24 samples, with 6.16–9.16 Gb data per sample and an error rate of <0.1%. Approximately 67.60%–75.31% and 66.71%–74.36% of these sequences were mapped to unique locations, whereas 0.89%–1.55% were mapped to multiple genome locations. A total of 44,248 predicted *B. rapa* genes were annotated. HTSeq (v0.6.1) was used to count the read numbers mapped to each gene, and the FPKM (fragments per kilobase of transcript sequence per million base pairs sequenced) was calculated based on the length of the gene and read counts mapped to this gene (Trapnell et al., 2010). Visualization of enrichment analysis was performed by the ggplot2 package (http://had.co.nz/ggplot2/).

Tests for pairwise differential expression were performed in the DESeq2 R package (Love et al., 2014). The resulting *p*-values were adjusted to control the false discovery rate (FDR), with genes having *p*-values < 0.05, |FoldChange| > 2, and FDR ≤ 0.01 considered to be differentially expressed genes (DEGs) for further analysis.

## 5 Conclusion

In this study, a total of 148 putative Chinese cabbage WRKY genes (BrWRKYs) were identified from the new Chinese cabbage genome (v3.0) (Chen et al., 2022), which may be potential resources for the development of *Brassica* varieties resistant against abiotic and biotic stresses. Time-series RNA-seq was carried out to elucidate the dynamic expression patterns of the BrWRKYs on the resistant mutant (sr) and the susceptible wild-type (inbred WT) challenged by Pc and revealed their transcriptional similarities and differences of WRKY family members in response to the pathogen. The findings improved our understanding of the WRKY-mediated soft rot stress response regulation in Chinese cabbage. The study thus lays a foundation for the genetic improvement of soft rot resistance.

## Data Availability

The datasets presented in this study can be found in online repositories. The names of the repository/repositories and accession number(s) can be found at: NCBI, GSE209906.
