# Supplementary figures and images for: Genome-wide identification and expression analysis of WRKY family genes under soft rot in Chinese cabbage
Source: Front Genet. 2022 Sep 26;13:958769. doi: 10.3389/fgene.2022.958769 (PMC9548547; doi:10.3389/fgene.2022.958769)

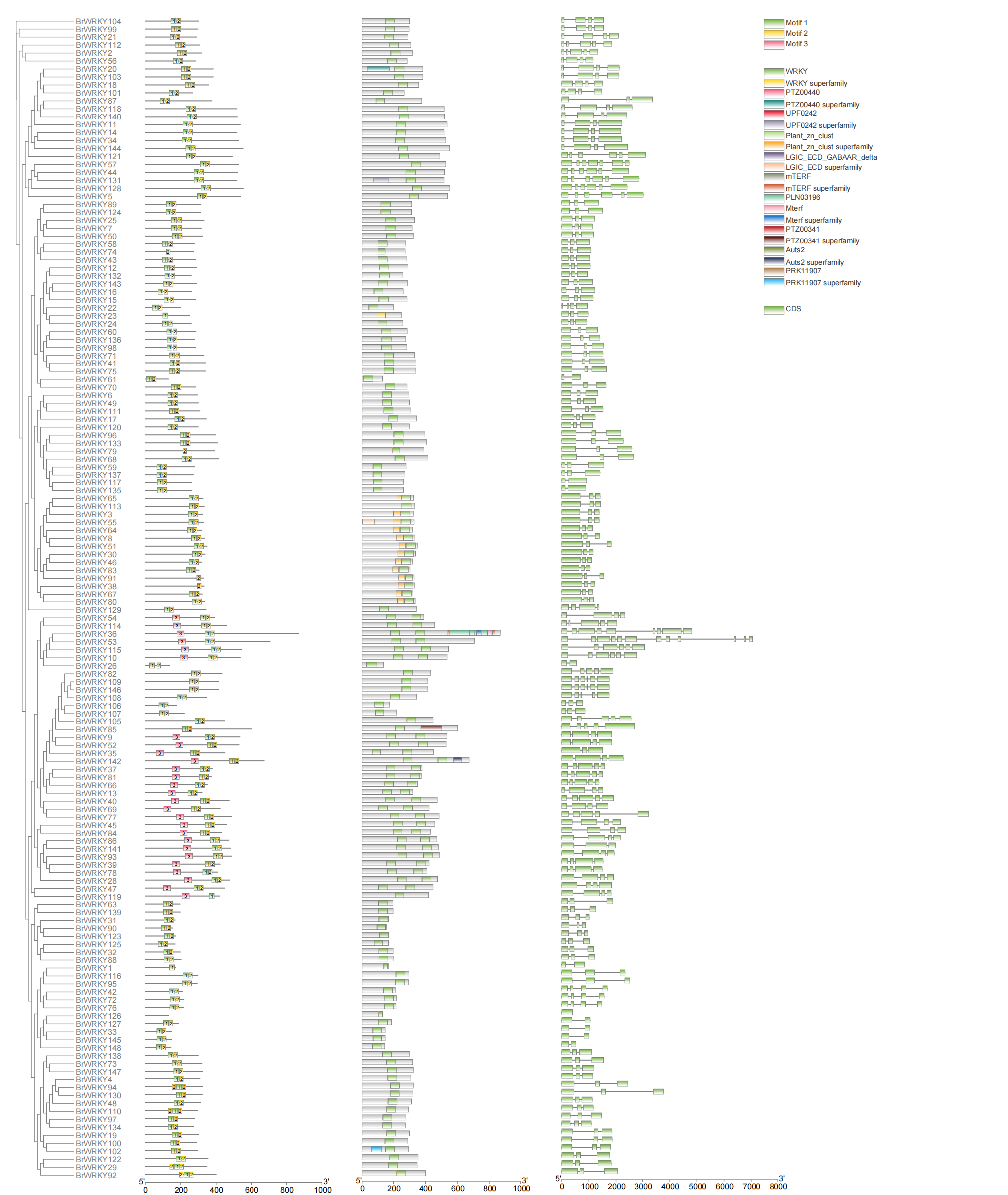

Supplement: Supplementary file 1 [file Image1.TIF]
